# Short-term effects of a multidisciplinary inpatient intensive rehabilitation treatment on body image in anorexia nervosa

**DOI:** 10.1186/s40337-023-00906-9

**Published:** 2023-10-06

**Authors:** Federico Brusa, Federica Scarpina, Ilaria Bastoni, Valentina Villa, Gianluca Castelnuovo, Emanuela Apicella, Sandra Savino, Leonardo Mendolicchio

**Affiliations:** 1 I.R.C.C.S. Istituto Auxologico Italiano, U.O. dei Disturbi del Comportamento Alimentare, Ospedale San Giuseppe, Piancavallo, VCO Italy; 2 I.R.C.C.S. Istituto Auxologico Italiano, Laboratorio di Psicologia, Ospedale San Giuseppe, Piancavallo, VCO Italy; 3 “Rita Levi Montalcini” Department of Neurosciences, University of Turin, Turin, Italy; 4 I.R.C.C.S. Istituto Auxologico Italiano, U.O. di Neurologia e Neuroriabilitazione, Ospedale San Giuseppe, Piancavallo, VCO Italy; 5 Psychology Department, Università Cattolica del Sacro Cuore, Milan, Italy

**Keywords:** Anorexia nervosa, Psychopathology, Psychological treatment, Body image

## Abstract

**Background:**

Positive changes in weight gain and eating pathology were reported after inpatient treatments for anorexia nervosa (AN). However, changes in the physical body do not always mirror changes in the imagined body. Here, the effect of a treatment focused on body image (BI) was described.

**Methods:**

This retrospective observational study had a quasi-experimental pre-post design without the control group. During the treatment, participants (N = 72) undertake a variety of activities focused on BI. The main outcome was tested through the Body Uneasiness Test.

**Results:**

At the end of the treatment, BI uneasiness decreased with a significant increase in weight gain.

**Conclusion:**

This study highlights the positive short-term effect of a multidisciplinary inpatient intensive rehabilitation treatment on BI in AN. We encourage to design of psychological treatments focusing on the cognitive and emotional bodily representation (i.e. the body in the mind) to increase physical well-being.

**Supplementary Information:**

The online version contains supplementary material available at 10.1186/s40337-023-00906-9.

## Abstract (plain language summary)

The sufferance of people affected by anorexia nervosa (AN) relies on different levels. Despite weight and eating habits being the most recognizable components of AN, people affected by AN can experience a very negative body image. The study highlights how a multidisciplinary inpatient intensive rehabilitation treatment for treating AN positively affects body image. Body image was targeted by specific activities, such as body image therapy and dance movement therapy. Rehabilitation programs should propose activities focusing on treating the cognitive and emotional bodily representation (i.e. the body in the mind) to increase physical well-being.

**Supplementary Information:**

The online version contains supplementary material available at 10.1186/s40337-023-00906-9.

## Background

Most of the inpatient treatments for anorexia nervosa (AN) for both adolescents and adults reported in the literature are designed to promote changes in weight gain and reduction of eating pathology [1, 2] since they are severely compromised in the disease [3, 4]. However, increased weight as well as improved eating habits do not always mirror positive changes in psychopathology, and sometimes affected people are resistant to changes [5–8]. One factor that may contribute to the efficacy of treatment is the negative body image (BI), meaning the complexity of beliefs, thoughts, perceptions, feelings, and behaviors towards the body [9, 10] typically observed in AN. Thus, this retrospective study aimed to verify the short-term effects of an inpatient intensive rehabilitation treatment for AN focused on BI.

## Methods

This retrospective observational study had a quasi-experimental pre-post design without the control group (for a similar approach see [11]). This study was approved by the Ethics Committee of the involved Institution (Reference number: 2022_11_22_05). All participants were volunteers who gave informed written consent before participating in this study; in the case of an age below 18 years old, parents signed the written consent. The Italian National Sanitary System covers all hospital charges and participants were not remunerated.

### Sample

Only in-patients consecutively recruited at their admission to the hospital between August 2021 and July 2022 (12 months) were included. Individuals were included in this study if (i) they were diagnosed with AN [3]; (ii) their body mass index (BMI) was ≤ 17 kg/m^2^ at the time of the admission (i.e. only moderate to extreme conditions [3]); (iii) they were compliant with the rehabilitation program; (iv) AN was the primary psychopathological diagnosis. Participants were excluded if (i) they withdrew from the program; (ii) other pathologies non-AN related were recorded (e.g. neurodegenerative diseases such as brain injury, and stroke); (iii) self-report questionnaires at admission or discharge were not completed. Notably, individuals in their first recovery and those in their second or more recovery were included: as the chronic nature of the disease [12], affected individuals frequently undertake more than one treatment.

### Multidisciplinary inpatient intensive rehabilitation treatment’s description

Individuals participated in a residential program for three to eight weeks (Table 1).

**Table 1:** List of activities proposed during the multidisciplinary inpatient intensive rehabilitation treatment

Activity	Frequency	Duration	Notes
Individual psychological consultation	1/week	45 min	Specialized psychologists provide support for psychological distress, and eating pathology
In-group psychotherapy	2/week	1 h	Specialized psychologists provide support on a group basis working on the disease, trauma, psychological distress, and eating pathology
Dance movement therapy	1/week	1 h	A specialized psychologist provides an intervention based on *Dance Movement Therapy* to improve BI perception
Body image therapy	2/week	30 min	Specialized physiotherapists provide interventions to work on BI distortion
Physiotherapy	5/week	30 min	Specialized physiotherapists provide tailored physiotherapy treatments
Low-intensity physical activities	3/week	30 min	Specialized physiotherapists provide gentle gymnastic sessions
Nutritional counseling—individual	1/week	30/45 min	Specialized dieticians provide dietary assessment, evaluation of nutrient intake and adequacy, nutritional status, anthropometric measures, eating patterns, and nutritional history
Nutritional counseling—group	1/week	1 h	Group intervention provided by a specialized dietician relative to healthy eating, general nutrition, and core food groups
Assisted meals	breakfast, snack, lunch, snack, dinner, snack	30 min to 1 h	Participants eat all together supervised by a dietician

The treatment is multidisciplinary, proposing both individual and in-group sessions focusing on the bodily experience; also, psychological functioning and nutrition are targeted. Body image difficulties were treated through BI therapy [13] and group dance therapy [14], since some previous evidence [15–17]. Also, low-intensity physical activities were proposed: individuals with AN sometimes experience the urge to move [18–20] and physical restlessness, with negative consequences on weight. Thus, physical movements were targeted as part of a healthy lifestyle.

Psychological functioning (e.g. depression, anxiety, and post-traumatic symptomatology) was targeted through both individual and in-group psychotherapy [21]. Finally, individual and in-group consultations with nutrition experts were delivered to identify tailored dietary schemes and to promote nutritional education about biological hunger, satiety, and nutrients [22]. Participants attended also assisted meals.

### Measures

All the psychological measures were completed at T0 (at the beginning of the treatment) and T1 (at the end of the rehabilitation program). The main outcome of the treatment (i.e. BI) was measured through the Body Uneasiness Test (BUT) [23], which assessed the individual level of discomfort towards their own body and BI. The questionnaire consists of 71 items, split into two parts with a six-point Likert scale (never, rarely, sometimes, often, usually, and always). Part A refers to weight phobia, body image concerns, avoidance, compulsive self-monitoring, detachment, and feelings of estrangement towards one's own body (i.e. depersonalization); part B measures specific worries about body parts or functions. The higher the score, the greater the body discomfort.

Moreover, as secondary outcomes, the pathological eating behavior through the Eating Disorder Inventory-3 (EDI-3) questionnaire [24], the psychological well-being through the Psychological General Well-Being Index questionnaire (PGWBI) [25], and the psychopathological components through the Symptom Check List-90 (SCL-90) [26] were measured. Details about these questionnaires (i.e. description and results) are reported in Additional file 1.

### Analyses

All the questionnaires (secondary outcomes included) were scored according to the seminal articles. A repeated measure analysis of covariance was used to highlight possible differences between the T0 and T1 (*Time*: T0 vs T1; main factor—within subjects) for each score, including delta (Δ) BMI (Δ*BMI*: T1-T0) as covariate to control the impact of BMI changes on the main within-subject factor of *Time*. Because of the high level of malnourishment, small variations in BMI may be observed over time. However, patients are very triggered by changes in their weight, which may have a not directly measurable effect on the outcomes. A paired sample t-test was used to verify any difference in the level of BMI registered at T0 and T1 within the two groups.

Pearson’s correlation was used to verify the relationship between the Δ score (Δ = T1-T0) of the *Global Severity Index* from BUT and the other outcomes tested (i.e. eating pathology by the *Eating Concerns Composite* scale (EDI-3), *Global score PGWBI* for the psychological well-being, and the *Obsessive–compulsive*, *Depression*, *Anxiety*, and *Global indices of distress* from the SCL-90 (other psychopathologies). Indeed, changes in BI may be related to changes in the other components treated in the intervention. Significant results were considered for *p* value ≤ .05 (two-tailed).

Because of ethical reasons, there is no restriction on age for admission to the rehabilitation program. Moreover, the length of recovery might differ because of the individual’s needs (i.e. tailoring approach). Therefore, in two supplementary analyses assessing, the main analysis relative to the main outcome of BI was run including these two factors as covariate (independently tested) Results are included respectively within Additional file 2 and Additional file 3.

## Results

### Participants

Overall, the information relative to 80 participants with AN was retrospectively inspected to be considered for this study; however, only 90% of participants were included, while 10% of the sample were excluded from the analyses because they dropped out from the rehabilitation program. The final dataset consisted of seventy-two participants with AN (type: 75% restrictive *vs* 25% binge-purging; 68 females; age: average (M) = 24.13, Standard Deviation (SD) = 12.43, min–max = 13–66); level of education: middle school N = 9, high school N = 51, bachelor’s degree N = 6, master’s degree N = 6; age at disease onset: M = 16.19, SD = 5.94, min–max = 10–50; disease length (from onset) in years: M = 7.93, SD = 10.76, min–max = 0–42). The mean of days of treatment was 36 (SD = 9 days). The mean level of BMI expressed as kg/m^2^ at the admission was 14.13 (SD = 1.58, min–max = 9.73–16.87*),* whereas at the discharge was 14.49 (SD = 1.45, min–max = 10.22–17.56): a significant increase after the treatment emerged (*t*(71) = 3.92; *p* < .001; d = .77).

### Main outcome: body image

For all the scales of BUT-A, scores from T0 to T1 (significant main effect of *Time*) decreased (Table 2), suggesting an amelioration in all the tested components. Also, the amelioration in the *Global Severity Index* and *Weight phobia* was still observed, also when controlling for the individual BMI variation ((a significant main effect of the covariate). No significant main effect of *Time* (no difference between scores at T0 and T1) or of the covariate (Table 2) were observed for the BUT – part B.

**Table 2:** Statistical results relative to the BUT, which is the main outcome of BI

	T0	T1	Statistical results Main effect time	Statistical results Covariate *Δ*BMI
*BUT-A*				
Global Severity Index	M = 3.05 SD = 1.03 Min–max = .44–4.79	M = 2.55 SD = .99 Min–max = 0.9–4.38	*F*(1,70) = 20.40; *p* < .001*; η^2^ = .23	*F*(1,70) = 4.46; *p* = .038*; η^2^ = .06
Weight phobia	M = 3.54 SD = 1.09 Min–max = .25–5	M = 3.08 SD = 1.12 Min–max = .13–4.88	*F*(1,70) = 11.33; *p* = .001*; η^2^ = .14	*F*(1,70) = 5.35; *p* = .024*; η^2^ = .07
Body image concern	M = 3.44 SD = 1.17 Min–max = .22–5	M = 2.85 SD = 1.19 Min–max = 0–5	*F*(1,70) = 21.45; *p* < .001*; η^2^ = .23	*F*(1,70) = 3.59; *p* = .062; η^2^ = .05
Avoidance	M = 2.32 SD = 1.15 Min–max = 0–4.67	M = 1.98 SD = 1.16 Min–max = 0–4.83	*F*(1,70) = 6.47; *p* = .013*; η^2^ = .08	*F*(1,70) = 2.03; *p* = .158; η^2^ = .03
Compulsive self-monitoring	M = 2.84 SD = 1.32 Min–max = 0–5	M = 2.36 SD = 1.13 Min–max = 0–5	*F*(1,70) = 15.36; *p* < .001*; η^2^ = .18	*F*(1,70) = 2.04; *p* = .158; η^2^ = .03
Depersonalization	M = 2.77 SD = 1.42 Min–max = 0–6.4	M = 2.09 SD = 1.24 Min–max = 0–4.8	*F*(1,70) = 22.56; *p* < .001*; η^2^ = .24	*F*(1,70) = 2.78; *p* = .100; η^2^ = .04
*BUT-B*				
Positive Symptom Total	M = 21.25 SD = 9.65 Min–max = 2–37	M = 20.86 SD = 10.53 Min–max = 0–37	*F*(1,70) = .84; *p* = .363; η^2^ = .01	*F*(1,70) = .47; *p* = .493; η^2^ = .01
Positive Symptom Distress Index	M = 3.10 SD = .72 Min–max = 1.46–5	M = 3.22 SD = 3.82 Min–max = 0–34.43	*F*(1,70) = .001; *p* = .972; η^2^ < .001	*F*(1,70) = .16; *p* = .688 η^2^ = .002

For some of the scales of the BUT-A, the main effect of *Time* was influenced by the covariate Δ*BMI* (corrected M and standard error of the mean (SE): Global Severity Index, M_T0_ = 3.06, SE_T0_ = .12, M_T1_ = 2.56, SE_T1_ = .12; Weight phobia, M_T0_ = 3.54, SE_T0_ = .13, M_T1_ = 3.08, SE_T1_ = .13). *Indicates a significant difference

### Secondary outcomes

As reported in Additional file 1, significant positive changes were observed also in eating pathology, psychological well-being, and more general psychological sufferance. This mirrored previous literature on the topic [27–30].

### Relationship between outcomes

As shown in Table 3, a significant positive relationship emerged between the Δ*Global Severity Index (BUT)*, and the Δ scores of the Δ*Eating Concerns Composite (EDI-3),* Δ*Obsessive-compulsive (SCL-90),* Δ*Depression (SCL-90)*, Δ*Anxiety (SCL-90)*, and Δ*Global indices of distress (SCL-90)*. Differently, a significant negative relationship between the Δ*Global Severity Index (BUT)* and the Δ*Global score PGWBI* was observed*.*

**Table 3:** The table reports the relationship between the Δ*Global Severity Index*, with the selected Δ from the other questionnaires

	Δ*Global Severity Index (BUT)*
Δ*Eating Concerns Composite (EDI-3)*	(*r* = .779, *n* = 72,* p* < .001*)
Δ*Global score PGWBI*	(*r* = -.531, *n* = 72,* p* < .001*)
Δ*Obsessive-compulsive (SCL-90)*	(*r* = .300, *n* = 72,* p* = .010*)
Δ*Depression (SCL-90)*	(*r* = .340, *n* = 72,* p* = .004*)
Δ*Anxiety (SCL-90)*	(*r* = .377, *n* = 72,* p* = .001*)
Δ*Global indices of distress (SCL-90)*	(*r* = .348, *n* = 72,* p* = .003*)

^*^Indicates a significant difference

Overall, the decrease in body uneasiness experienced after the treatment was significantly related to positive changes in terms of eating pathology, psychological stress (i.e. obsessive–compulsive, depressive, and anxiety symptoms) as well as psychological well-being.

### The role of age and length of recovery

As reported in the Supplementary materials, the main effect of the treatment on BUT was still significant, also when controlling for the covariate age; differently, we did not observe a significant main effect of the treatment when controlling for the length of recovery.

## Discussion

The aim of the current study was to verify the short-term within-group effects of a multidisciplinary inpatient intensive rehabilitation treatment on BI in a sample of individuals affected by AN.

After the treatment, changes in BI were observed, as suggested by the positive increase in the scores relative to all the components of BUT—part A [23], together with decreased psychopathological eating symptoms and increased psychological well-being (see Additional file 1). The multidisciplinary treatment includes activities designed to mitigate body misperception in AN, fostering the acceptance of bodily changes and the decrease of cognitive bodily distortions [31] through multiple positive experiences involving body actions: patients may perceive positive sensorimotor feedback from their own body when engaged in imagined and physical movements.

Notably, it was not observe any changes for part B of BUT, which measures specific worries about particular body parts or functions. The rehabilitation activities were not focused on single body parts, since it was aimed to help participants experience the body as a whole, to decrease self-monitoring and checking behavior [32, 33]. Together with a more positive BI perception, the physical body changed in participants with an increased BMI after the treatment. It may be expected that BI would be negatively influenced by weight gain [7, 8]: individuals with AN would be highly worried about seeing weight increase. This may be not the case, since changes in the physical body were sustained by positive changes in cognitive and emotional bodily representation (i.e. BI) [23]. The results about the physical body and imagined body recall the concept according to which the body cannot be dissociated from the mind, and vice versa [34]. Both components are crucial in the individual bodily experience, specifically in the case of illness: AN does not concern only the physical body, but also how the body is mentally thought and represented [35]. This is something that should be taken more into account in designing rehabilitative treatments for AN [36, 37], as here proposed.

The current study presents some limitations. Here, it was a monocentric-based study, as done elsewhere [1, 29, 30]; therefore, such results should be generalized with some caution. A control group (i.e. not-treated affected individuals) was not included because of ethical reasons [11, 38]: during hospitalization, treatments must be guaranteed for each patient. Only self-report measurements, even though they are highly used in clinical and research contexts, were used as outcomes. Lastly, only people compliant with the rehabilitation program were included in the sample: it was recorded that approximately 10% of patients dropped out of the program. Drop-out from inpatient programs is something well-known in the literature [39]; however, the reasons for the drop-out (i.e., noncompliance, disease worsening requiring access to the emergency room, or other external factors) and when participants retired from the program are generally not recorded. However, what factors may anticipate the drop-out should be analyzed to increase the efficacy of the treatment and maximize the inclusion of participants in the program.

## Conclusions

The study highlighted the positive short-term effect of a multidisciplinary inpatient intensive rehabilitation treatment on BI, together with increased BMI and amelioration of eating pathology and psychological well-being, in individuals with AN. The results of the study may encourage to design psychological treatments focusing on BI, even in the case of very low BMI: restoring a positive cognitive and emotional bodily representation (the body in mind) may increase physical and psychological well-being.

### Supplementary Information

**Additional file 1**. Supplementary analyses-secondary outcomes.

**Additional file 2**. Supplementary analyses-covariate: age.

**Additional file 3**. Supplementary analyses-covariate: length of recovery.

## Data Availability

The data that support the findings of this study are available on reasonable request in Zenodo at: https://zenodo.org/record/8297953.

## Abbreviations

AN: Anorexia nervosa
BI: Body image
BMI: Body mass index
BUT: Body uneasiness test
EDI-3: Eating disorder inventory-3
M: Average
PGWBI: Psychological general well-being index
SCL-90: Symptom check list-90
SD: Standard deviation
SE: Standard error of the mean
