# Correction: Short-term effects of a multidisciplinary inpatient intensive rehabilitation treatment on body image in anorexia nervosa

**DOI:** 10.1186/s40337-024-01072-2

**Published:** 2024-08-05

**Authors:** Federico Brusa, Federica Scarpina, Ilaria Bastoni, Valentina Villa, Gianluca Castelnuovo, Emanuela Apicella, Sandra Savino, Leonardo Mendolicchio

**Affiliations:** 1 I.R.C.C.S. Istituto Auxologico Italiano, U.O. dei Disturbi del Comportamento Alimentare, Ospedale San Giuseppe, Piancavallo, VCO Italy; 2 I.R.C.C.S. Istituto Auxologico Italiano, Laboratorio di Psicologia, Ospedale San Giuseppe, Piancavallo, VCO Italy; 3 “Rita Levi Montalcini” Department of Neurosciences, University of Turin, Turin, Italy; 4 I.R.C.C.S. Istituto Auxologico Italiano, U.O. di Neurologia e Neuroriabilitazione, Ospedale San Giuseppe, Piancavallo, VCO Italy; 5 Psychology Department, Università Cattolica del Sacro Cuore, Milan, Italy

**Correction to: Federico et al. Journal of Eating Disorders (2023) 11:178**

10.1186/s40337-023-00906-9

Following the publication of the Original Article, the authors identified that the given names and surnames for all the authors were switched as follows: Brusa Federico, Scarpina Federica, Bastoni Ilaria, Villa Valentina, Castelnuovo Gianluca, Apicella Emanuela, Savino Sandra and Mendolicchio Leonardo. Consequently, in the Author Contributions section, the authors' initials were also mistakenly switched.

The author group has been updated above and the Original Article has been corrected.

