# Supplementary material for: Short-term effects of a multidisciplinary inpatient intensive rehabilitation treatment on body image in anorexia nervosa
Source: J Eat Disord. 2023 Oct 6;11:178. doi: 10.1186/s40337-023-00906-9 (PMC10559592; doi:10.1186/s40337-023-00906-9)
Supplement: Supplementary file 1 — Additional file 1. Supplementary analyses-secondary outcomes. [file 40337_2023_906_MOESM1_ESM.docx]

**Short-term effects of a** **multidisciplinary inpatient** **intensive rehabilitation treatment on body image in anorexia nervosa.**

Brusa Federico^a,b^, Scarpina Federica^c,d^, Bastoni Ilaria^a,b^, Villa Valentina^a,b^, Castelnuovo Gianluca^b,e^, Apicella Emanuela^a^, Savino Sandra^a^, Mendolicchio Leonardo^a^

^a^ I.R.C.C.S. Istituto Auxologico Italiano, U.O. dei Disturbi del Comportamento Alimentare, Ospedale San Giuseppe, Piancavallo (VCO), Italy

^b^ I.R.C.C.S. Istituto Auxologico Italiano, Laboratorio di Psicologia, Ospedale San Giuseppe, Piancavallo (VCO), Italy

^c^ “Rita Levi Montalcini” Department of Neurosciences, University of Turin, Italy

^d^ I.R.C.C.S. Istituto Auxologico Italiano, U.O. di Neurologia e Neuroriabilitazione, Ospedale San Giuseppe, Piancavallo (VCO), Italy

^e^ Psychology Department, Università Cattolica del Sacro Cuore, Milan, Italy

**Corresponding author**

Brusa Federico: [f.brusa@auxologico.it](mailto:f.brusa@auxologico.it); I.R.C.C.S. Istituto Auxologico Italiano, U.O. dei Disturbi del Comportamento Alimentare, Ospedale San Giuseppe, Piancavallo (VCO), Italy; I.R.C.C.S. Istituto Auxologico Italiano, Laboratorio di Psicologia, Ospedale San Giuseppe, Piancavallo (VCO), Italy

**Additional file 1**

***Secondary outcomes.***

*Eating pathology.*

The pathological eating behavior was assessed using the Eating Disorder Inventory-3 (EDI-3) questionnaire [1]. The questionnaire consists of 91 items evaluated on a six-point Likert scale (always, usually, often, sometimes, rarely, never). The 91 items are divided into twelve primary scales, about which three refer specifically to the eating disorder (drive for thinness, bulimia, body dissatisfaction) and nine relatives to general psychological scales relevant but non-specific for eating disorders (low self-esteem, personal alienation, interpersonal insecurity, interpersonal alienation, interoceptive deficits, emotional dysregulation, perfectionism, asceticism, maturity fears); also, there are six composite scales (one eating-disorder-specific and five relevant but non-specific for eating disorders: eating concerns composite, ineffectiveness composite, interpersonal problems composite, affective problems composite, overcontrol composite, global psychological maladjustment). The higher the score, the greater the severity of the symptoms.

*Psychological well-being.*

The effect of the intensive rehabilitation treatment on the psychological well-being of participants was assessed through the Psychological General Well-Being Index (PGWBI) [2] as done in other studies [3,4,5]. The questionnaire consists of 22 items, rated on a six-point Likert scale (0 to 5, with the sentences corresponding to values changing concerning the specific item), measuring the domains of anxiety, depressed mood, positive well-being, self-control, general health, and vitality. The questionnaire has an overall score ranging from 0 to 110 points. The higher the score, the higher the subjective perception of psychological well-being.

*Psychopathological comorbidities.*

The SCL-90 questionnaire [6] consists of 90 items with a five-point Likert scale (not at all, a little bit, moderately, quite a bit, extremely) that describe nine symptomatologic dimensions: Somatization; Obsessive-Compulsive; Interpersonal Sensitivity; Depression; Anxiety; Hostility; Phobic Anxiety; Paranoid Ideation; and Psychoticism. A global score can also be calculated, the SCL90 Global Severity Index which measures the general psychopathological state. When the average score is greater than or equal to 1, this is an index of significant psychopathology. Overall, the higher the score, the higher the psychopathology.

***Results***

*Eating pathology.*

For all the scales composing the EDI-3 questionnaire, we observed a decrease in the scores from T0 to T1 (significant main effect of *Time*), suggesting a decrease in the manifestation of the eating-related symptomatology. Such an effect was not influenced by the covariate Δ*BMI* in most cases (Table 1), with some exceptions, which are Drive For Thinness, Low Self-Esteem, Personal Alienation, Interoceptive Deficits, Eating Concerns Composite, and Ineffectiveness Composite. For these scores, we observed a main effect of this covariate on the main statistical difference, suggesting a role of the BMI variation on the treatment outcomes for these specific components.

|  | **T0** | **T1** | **Statistical Results Main Effect Time** | **Statistical Results Covariate** Δ **BMI** |
| --- | --- | --- | --- | --- |
| **Eating Disorder Specific Scales** | | | | |
| **Drive For Thinness** | M = 22.29  SD = 7.45  min-max = 0-28 | M = 17.01  SD = 8.53  min-max = 0-28 | *F*(1,70) = 28.56;  p < .001*****;  η^2^ = .29 | *F*(1,70) = 6.19;  p = .015*****;  η^2^ = .08 |
| **Bulimia** | M = 5.63  SD = 7.83  min-max = 0-32 | M = 3.75  SD = 5.79  min-max = 0-28 | *F*(1,70) = 6.30;  p = .014*****;  η^2^ = .08 | *F*(1,70) = 1.62;  P = .208;  η^2^ = .02 |
| **Body Dissatisfaction** | M = 28.15  SD = 7.25  min-max = 15-40 | M = 23.85  SD = 8.75  min-max = 4-40 | *F*(1,70) = 26.30;  p < .001*****;  η^2^ = .27 | *F*(1,70) = 3.52;  p = .065;  η^2^ = .06 |
| **Psychological Trait Scales** | | | | |
| **Low Self-Esteem** | M = 15.93  SD = 5.84  min-max = 1-24 | M = 12.86  SD = 6.06  min-max = 0-24 | *F*(1,70) = 25.72;  p < .001*****;  η^2^ = .27 | *F*(1,70) = 7.61;  p = .007*****;  η^2^ = .10 |
| **Personal Alienation** | M = 15.24  SD = 6.56  min-max = 2-28 | M = 12.43  SD = 6.38  min-max = 1-27 | *F*(1,70) = 25.91;  p < .001*****;  η^2^ = .27 | *F*(1,70) = 4.96;  p = .029*****;  η^2^ = .07 |
| **Interpersonal Insecurity** | M = 13.53  SD = 6.82  min-max = 0-26 | M = 11.72  SD = 6.38  min-max = 0-26 | *F*(1,70) = 14.75;  p < .001*****;  η^2^ = .17 | *F*(1,70) = .03;  P = .860;  η^2^ < .001 |
| **Interpersonal Alienation** | M = 12.85  SD = 5.83  min-max = 1-28 | M = 10.99  SD = 5.55  min-max = 1-23 | *F*(1,70) = 21.50;  p < .001*****;  η^2^ = .23 | *F*(1,70) = 1.02;  p = .317;  η^2^ = .01 |
| **Interoceptive Deficits** | M = 21.28  SD = 8.50  min-max = 2-36 | M = 16.61  SD = 8.35  min-max = 0-36 | *F*(1,70) = 30.06;  p < .001*****;  η^2^ = .30 | *F*(1,70) = 4.69;  p = .034*****;  η^2^ = .06 |
| **Emotional Dysregulation** | M = 10.89  SD = 7.20  min-max = 0-37 | M = 8.13  SD = 5.93  min-max = 0-25 | *F*(1,70) = 14.02;  p < .001*****;  η^2^ = .17 | *F*(1,70) = 1.25;  P = .267;  η^2^ = .02 |
| **Perfectionism** | M = 11.76  SD = 5.10  min-max = 0-24 | M = 10.82  SD = 5.45  min-max = 0-24 | *F*(1,70) = 6.10;  p = .016*****;  η^2^ = .08 | *F*(1,70) = .58;  p = .449;  η^2^ = .01 |
| **Ascetism** | M = 14.36  SD = 5.78  min-max = 4-28 | M = 11.54  SD = 6.33  min-max = 0-28 | *F*(1,70) = 21.54;  p < .001*****;  η^2^ = .24 | *F*(1,70) = 2.81;  p = .098;  η^2^ = .04 |
| **Maturity Fears** | M = 17.31  SD = 7.34  min-max = 0-32 | M = 15.64  SD = 7.14  min-max = 0-32 | *F*(1,70) = 9.27;  p = .003*****;  η^2^ = .12 | *F*(1,70) = .23;  p = .636;  η^2^ = .003 |
| **Composite Scales** | | | | |
| **Eating Concerns Composite** | M = 56.07  SD = 16.48  min-max = 20-96 | M = 44.61  SD = 18.68  min-max = 5-93 | *F*(1,70) = 32.52;  p < .001*****;  η^2^ = .32 | *F*(1,70) = 6.35;  p = .014*****;  η^2^ = .08 |
| **Ineffectiveness Composite** | M = 31.17  SD = 11.84  min-max = 5-52 | M = 25.29  SD = 11.93  min-max = 4-49 | *F*(1,70) = 29.79;  p < .001*****;  η^2^ = .30 | *F*(1,70) = 6.68;  p = .012*****;  η^2^ = .09 |
| **Interpersonal Problems Composite** | M = 26.38  SD = 11.65  min-max = 3-51 | M = 22.71  SD = 10.76  min-max = 3-48 | *F*(1,70) = 24.70;  p < .001*****;  η^2^ = .26 | *F*(1,70) = .37;  p = .544;  η^2^ = .01 |
| **Affective Problems Composite** | M = 32.03  SD = 13.69  min-max = 2-63 | M = 24.60  SD = 13.05  min-max = 0-54 | *F*(1,70) = 32.76;  p < .001*****;  η^2^ = .32 | *F*(1,70) = 3.61;  p = .062;  η^2^ = .05 |
| **Overcontrol Composite** | M = 26.13  SD = 9.29  min-max = 5-51 | M = 22.36  SD = 9.83  min-max = 0-52 | *F*(1,70) = 19.10;  p < .001*****;  η^2^ = .21 | *F*(1,70) = 2.20;  p = .143;  η^2^ = .03 |
| **Global Psychological Maladjustment** | M = 133.00  SD = 40.04  min-max = 46-237 | M = 110.74  SD = 41.85  min-max = 21-210 | *F*(1,70) = 38.57;  p < .001*****;  η^2^ = .36 | *F*(1,70) = 3.18;  p = .079;  η^2^ = .04 |

**Table 1:** The table reports the statistics of the EDI-3 questionnaire. For some of the scales, the main effect of *Time* was influenced by the covariate Δ*BMI* (corrected M and SE: Drive For Thinness, M_T0_ = 22.29, SE_T0_ = .86, M_T1_ = 17.01, SE_T1_ = .98; Low Self-Esteem, M_T0_ = 15.93, SE_T0_ = .65, M_T1_ = 12.86, SE_T1_ = .70; Personal Alienation, M_T0_ = 15.24, SE_T0_ = .73, M_T1_ = 12.43, SE_T1_ = .75; Interoceptive Deficits, M_T0_ = 21.28, SE_T0_ = .97, M_T1_ = 16.61, SE_T1_ = .98; Eating Concerns Composite, M_T0_ = 56.07, SE_T0_ = 1.86, M_T1_ = 44.61, SE_T1_ = 2.16; Ineffectiveness Composite, M_T0_ = 31.17, SE_T0_ = 1.31, M_T1_ = 25.29, SE_T1_ = 1.40). ***** Indicated a significant difference.

*Psychological well-being.*

For all the scales composing the PGWBI questionnaire, we observed a significant increase in the scores from T0 to T1 (significant main effect of *Time*), suggesting an amelioration of psychological well-being. Such an effect was not influenced by the covariate Δ*BMI* (Table 2), suggesting that the changes in the BMI did not influence the changes in psychological well-being.

|  | **T0** | **T1** | **Statistical Results**  **main effect Time** | **Statistical Results**  **Covariate** Δ **BMI** |
| --- | --- | --- | --- | --- |
| **Anxiety** | M = 9.44  SD = 5.31  min-max = 0-24 | M = 13.94  SD = 5.32  min-max = 2-25 | *F*(1,70) = 51.27;  p < .001*****;  η^2^ = .42 | *F*(1,70) = 2.45;  p = .122;  η^2^ = .03 |
| **Depression** | M = 6.78  SD = 4.25  min-max = 0-14 | M = 9.72  SD = 3.33  min-max = 1-15 | *F*(1,70) = 42.73;  p < .001*****;  η^2^ = .38 | *F*(1,70) = .15;  p = .698;  η^2^ = .002 |
| **Positive well-being** | M = 4.97  SD = 3.31  min-max = 0-15 | M = 8.51  SD = 3.96  min-max = 0-19 | *F*(1,70) = 51.98;  p < .001*****;  η^2^ = .43 | *F*(1,70) = 2.01;  p = .160;  η^2^ = .03 |
| **Self-control** | M = 6.56  SD = 3.28  min-max = 0-15 | M = 9.21  SD = 3.10  min-max = 1-15 | *F*(1,70) = 52.97  p < .001*****;  η^2^ = .43 | *F*(1,70) = 1.01  p = .316;  η^2^ = .01 |
| **General Health** | M = 7.89  SD = 3.09  min-max = 0-14 | M = 9.61  SD = 2.71  min-max = 1-14 | *F*(1,70) = 14.36;  p < .001*****;  η^2^ = .17 | *F*(1,70) = .15;  p = .694;  η^2^ = .002 |
| **Vitality** | M = 7.42  SD = 4.36  min-max = 0-19 | M = 11.67  SD = 4.25  min-max = 1-19 | *F*(1,70) = 55.22;  p < .001*****;  η^2^ = .44 | *F*(1,70) = .01;  p = .921;  η^2^ = < .001 |
| **Total Score** | M = 43.24  SD = 18.74  min-max = 5-87 | M = 62.65  SD = 18.98  min-max = 16-102 | *F*(1,70) = 65.83;  p < .001*****;  η^2^ = .49 | *F*(1,70) = 1.12;  p = .294;  η^2^ = .02 |

**Table 2:** The table reports the statistics of the PGWBI questionnaire. ***** Indicated a significant difference.

*Psychopathological comorbidities.*

For most of the scales composing the SCL-90, we observed a decrease in the scores from T0 to T1 (significant main effect of *Time*), suggesting a decrease in the not eating-related symptomatology, with an exception for the scales relative to the level of hostility and psychoticism, about which there was not a significant main effect of *Time* (i.e. no difference between scores at T0 and T1) (Table 3). The main within-group effect of *Time* was influenced by the covariate Δ*BMI* in the case of Interpersonal sensitivity, Depression, Paranoid ideation, and Global indices of distress (Table 3).

|  | **T0** | **T1** | **Statistical Results**  **Main effect Time** | **Statistical Results**  **Covariate** Δ **BMI** |
| --- | --- | --- | --- | --- |
| **Somatization** | M = 1.70  SD = .96  min-max = 0-3.92 | M = 1.39  SD = .86  min-max = .17-3.17 | *F*(1,70) = 13.34;  p < .001*****;  η^2^ = .16 | *F*(1,70) = 1.71;  p = .195;  η^2^ = .02 |
| **Obsessive-compulsive** | M = 2.12  SD = .86  min-max = .2-3.9 | M = 1.83  SD = .88  min-max = .06-3.6 | *F*(1,70) = 11.30;  p = .001*****;  η^2^ = .14 | *F*(1,70) = 1.64;  p = .205;  η^2^ = .02 |
| **Interpersonal sensitivity** | M = 2.14  SD = .92  min-max = .22-3.89 | M = 1.91  SD = .92  min-max = .22-3.56 | *F*(1,70) = 7.23;  p = .009*****;  η^2^ = .09 | *F*(1,70) = 5.08;  p = .027*****;  η^2^ = .07 |
| **Depression** | M = 2.44  SD = .86  min-max = 0-3.92 | M = 2.20  SD = .89  min-max = .38-3.69 | *F*(1,70) = 6.76;  p = 011*****;  η^2^ = .09 | *F*(1,70) = 6.39;  p = .014*****;  η^2^ = .08 |
| **Anxiety** | M = 2.08  SD = .91  min-max = 0-3.7 | M = 1.73  SD = .87  min-max = .1-3.9 | *F*(1,70) = 17.31;  p < .001*****;  η^2^ = .20 | *F*(1,70) = 3.91;  p = .052;  η^2^ = .05 |
| **Hostility** | M = 1.20  SD = .91  min-max = 0-3.67 | M = 1.04  SD = .84  min-max = 0-4 | *F*(1,70) = 1.92;  p = .170;  η^2^ = .03 | *F*(1,70) = 6.53;  p = .013*****;  η^2^ = .08 |
| **Phobic anxiety** | M = 1.09  SD = .85  min-max = 0-3 | M = .88  SD = .83  min-max = 0-2.86 | *F*(1,70) = 9.79;  p = .003*****;  η^2^ = .12 | *F*(1,70) = .78;  p = .381;  η^2^ = .01 |
| **Psychoticism** | M = 1.29  SD = .66  min-max = 0-3 | M = 1.20  SD = .64  min-max = 0-2.5 | *F*(1,70) = .30;  p = .086;  η^2^ = .04 | *F*(1,70) = 1.47;  p = .229;  η^2^ = .02 |
| **Paranoid ideation** | M = 1.85  SD = .83  min-max = 0-3.67 | M = 1.67  SD = .87  min-max = 0-4 | *F*(1,70) = 4.13;  p = .046*****;  η^2^ = .06 | *F*(1,70) = 4.43;  p = .039*****;  η^2^ = .06 |
| **Global indices of distress** | M = 1.84  SD = .71  min-max = .13-3.19 | M = 1.60  SD = .68  min-max = .27-2.98 | *F*(1,70) = 6.35;  p = .014*****;  η^2^ = .08 | *F*(1,70) = 10.66;  p = .002*****;  η^2^ = .13 |

**Table 3:** the table reports the statistic of the SCL-90 questionnaire (corrected M and SE: Interpersonal sensitivity, M_T0_ = 2.14, SE_T0_ = .11, M_T1_ = 1.91, SE_T1_ = .11; Depression, M_T0_ = 2.44, SE_T0_ = .10, M_T1_ = 2.20, SE_T1_ = .10; Paranoid ideation, M_T0_ = 1.86, SE_T0_ = .09, M_T1_ = 1.68, SE_T1_ = .10; Global indices of distress, M_T0_ = 2.24, SE_T0_ = .40, M_T1_ = 1.61, SE_T1_ = .08). ***** Indicated a significant difference.

**References**

1. Clausen L, Rosenvinge JH, Friborg O, Rokkedal K. Validating the eating disorder inventory-3 (EDI-3): A comparison between 561 female eating disorders patients and 878 females from the general population. Journal of Psychopathology and Behavioral Assessment. 2010;33(1):101–10.
2. Dupuy HJ. “The Psychological General Well-Being (PGWB) Index,” in Assessment of Quality of Life in Clinical Trials of Cardiovascular Therapies. In: Wenger N editor. New York: Le Jacq1984. 170–83.
3. Scarpina F, Bastoni I, Cappelli S, Priano L, Giacomotti E, Castelnuovo G, et al. Short-term effects of a multidisciplinary residential rehabilitation program on perceived risks, confidence toward continuous positive airway pressure treatment, and self-efficacy in a sample of individuals affected by obstructive sleep apnea syndrome. Frontiers in Psychology. 2021;12.
4. Lundgren-Nilsson Å, Jonsdottir IH, Ahlborg G, Tennant A. Construct validity of the Psychological General Well Being Index (PGWBI) in a sample of patients undergoing treatment for stress-related exhaustion: A Rasch analysis. Health and Quality of Life Outcomes. 2013;11(1).
5. Manzoni GM, Villa V, Compare A, Castelnuovo G, Nibbio F, Titon AM, et al. Short-term effects of a multi-disciplinary cardiac rehabilitation program on psychological well-being, exercise capacity and weight in a sample of obese in-patients with coronary heart disease: A practice-level study. Psychology, Health & Medicine. 2011;16(2):178–89.
6. Sarno I, Preti E, Prunas A, Madeddu F. SCL-90-R. Symptom checklist-90-R. Giunti Organizzazioni Speciali: Firenze; 2011.
