# Supplementary material for: Short-term effects of a multidisciplinary inpatient intensive rehabilitation treatment on body image in anorexia nervosa
Source: J Eat Disord. 2023 Oct 6;11:178. doi: 10.1186/s40337-023-00906-9 (PMC10559592; doi:10.1186/s40337-023-00906-9)
Supplement: Supplementary file 2 — Additional file 2. Supplementary analyses-covariate: age. [file 40337_2023_906_MOESM2_ESM.docx]

**Short-term effects of a** **multidisciplinary inpatient** **intensive rehabilitation treatment on body image in anorexia nervosa.**

Brusa Federico^a,b^, Scarpina Federica^c,d^, Bastoni Ilaria^a,b^, Villa Valentina^a,b^, Castelnuovo Gianluca^b,e^, Apicella Emanuela^a^, Savino Sandra^a^, Mendolicchio Leonardo^a^

^a^ I.R.C.C.S. Istituto Auxologico Italiano, U.O. dei Disturbi del Comportamento Alimentare, Ospedale San Giuseppe, Piancavallo (VCO), Italy

^b^ I.R.C.C.S. Istituto Auxologico Italiano, Laboratorio di Psicologia, Ospedale San Giuseppe, Piancavallo (VCO), Italy

^c^ “Rita Levi Montalcini” Department of Neurosciences, University of Turin, Italy

^d^ I.R.C.C.S. Istituto Auxologico Italiano, U.O. di Neurologia e Neuroriabilitazione, Ospedale San Giuseppe, Piancavallo (VCO), Italy

^e^ Psychology Department, Università Cattolica del Sacro Cuore, Milan, Italy

**Corresponding author**

Brusa Federico: [f.brusa@auxologico.it](mailto:f.brusa@auxologico.it); I.R.C.C.S. Istituto Auxologico Italiano, U.O. dei Disturbi del Comportamento Alimentare, Ospedale San Giuseppe, Piancavallo (VCO), Italy; I.R.C.C.S. Istituto Auxologico Italiano, Laboratorio di Psicologia, Ospedale San Giuseppe, Piancavallo (VCO), Italy

**Additional file 2**

Here, we report the results on body image when the role of age was considered. More in detail, a repeated measure analysis of covariance was used to highlight possible differences between the T0 and T1 (Time: T0 vs T1; main factor – within subjects). The covariate factor was *AGE*.

For most of the scales composing the Body Uneasiness Test (BUT) part A (*Global Severity Index*, *Weight phobia*, *Body image concern*, and *Depersonalization*), we observed a decrease in the scores from T0 to T1 (significant main effect of *Time*) (Table 1), suggesting an improved body image’s perception. We also observed a main effect of the covariate *AGE* on the main statistical difference for the scales *Global Severity Index*, *Weight phobia*, *Body image concern*, and *Compulsive self-monitoring* suggesting that the age variation was linked to the changes in these treatment outcomes. Differently, for the scales composing the BUT part B, we did not observe a significant main effect of *Time* (no difference between scores at T0 and T1), nor did we observe a main effect of the covariate *AGE* on the main statistical difference (Table 1).

|  | **T0** | **T1** | **Statistical Results**  **main effect Time** | **Statistical Results**  **Covariate AGE** |
| --- | --- | --- | --- | --- |
| **BUT-A** | | | | |
| **Global Severity Index** | M = 3.05  SD = 1.03  min-max = .44-4.79 | M = 2.55  SD = .99  min-max = 0.9-4.38 | *F*(1,70) = 8.65;  p = .004*****;  η^2^ = .11 | *F*(1,70) = 14.51;  p < .001*****;  η^2^ = .17 |
| **Weight phobia** | M = 3.54  SD = 1.09  min-max = .25-5 | M = 3.08  SD = 1.12  min-max = .13-4.88 | *F*(1,70) = 6.44;  p = .013*****;  η^2^ = .08 | *F*(1,70) = 16.47;  p < .001*****;  η^2^ = .19 |
| **Body image concern** | M = 3.44  SD = 1.17  min-max = .22-5 | M = 2.85  SD = 1.19  min-max = 0-5 | *F*(1,70) = 9.32;  p = .003*****;  η^2^ = .12 | *F*(1,70) = 12.37;  p = < .001*****;  η^2^ = .15 |
| **Avoidance** | M = 2.32  SD = 1.15  min-max = 0-4.67 | M = 1.98  SD = 1.16  min-max = 0-4.83 | *F*(1,70) = 2.09;  p = .152**;**  η^2^ = .03 | *F*(1,70) = 3.78;  p = .056;  η^2^ = .05 |
| **Compulsive self-monitoring** | M = 2.84  SD = 1.32  min-max = 0-5 | M = 2.36  SD = 1.13  min-max = 0-5 | *F*(1,70) = 3.09;  p = .083;  η^2^ = .04 | *F*(1,70) = 16.87;  p < .001*;  η^2^ = .19 |
| **Depersonalization** | M = 2.77  SD = 1.42  min-max = 0-6.4 | M = 2.09  SD = 1.24  min-max = 0-4.8 | *F*(1,70) = 12.80;  p < .001*****;  η^2^ = .16 | *F*(1,70) = 3.56;  p = .063;  η^2^ = .05 |
| **BUT-B** | | | | |
| **Positive Symptom Total** | M = 21.25  SD = 9.65  min-max = 2-37 | M = 20.86  SD = 10.53  min-max = 0-37 | *F*(1,70) = 2.65;  p = .108;  η^2^ = .04 | *F*(1,70) = 14.10;  p < .001*;  η^2^ = .17 |
| **Positive Symptom Distress Index** | M = 3.10  SD = .72  min-max = 1.46-5 | M = 3.22  SD = 3.82  min-max = 0-34.43 | *F*(1,70) = 1.701;  p = .196;  η^2^ = .02 | *F*(1,70) = .92;  p = .341  η^2^ = .01 |

**Table 1:** The table reports the statistics of the BUT questionnaire. For some of the scales of the questionnaire, the main effect of *Time* was influenced by the covariate *AGE* (corrected M and SE: Global Severity Index, M_T0_ = 3.06, SE_T0_ = .11, M_T1_ = 2.56, SE_T1_ = .11; Weight phobia, M_T0_ = 3.54, SE_T0_ = .12, M_T1_ = 3.08, SE_T1_ = .13; Body image concern, M_T0_ = 3.45, SE_T0_ = .13, M_T1_ = 2.86, SE_T1_ = .14; Compulsive self-monitoring, M_T0_ = 2.84, SE_T0_ = .15, M_T1_ = 2.36, SE_T1_ = .12; Positive Symptom Total, M_T0_ = 21.25, SE_T0_ = 1.01, M_T1_ = 20.86, SE_T1_ = 1.20). ***** Indicated a significant difference.
